# Supplementary material for: Crimean–Congo haemorrhagic fever virus uses LDLR to bind and enter host cells
Source: Nat Microbiol. 2024 Mar 28;9(6):1499–512. doi: 10.1038/s41564-024-01672-3 (PMC11153131; doi:10.1038/s41564-024-01672-3)
Supplement: Supplementary file 1 — Reporting Summary [file 41564_2024_1672_MOESM1_ESM.pdf]

nature portfolio

Corresponding author(s): Ali Mirazimi
Joseph Penninger

Last updated by author(s): Feb 25, 2024

Reporting Summary

Nature Portfolio wishes to improve the reproducibility of the work that we publish. This form provides structure for consistency and transparency in reporting. For further information on Nature Portfolio policies, see our Editorial Policies and the Editorial Policy Checklist.

Statistics

For all statistical analyses, confirm that the following items are present in the figure legend, table legend, main text, or Methods section.

n/a	Confirmed	
☐	☒	The exact sample size (*n*) for each experimental group/condition, given as a discrete number and unit of measurement
☐	☒	A statement on whether measurements were taken from distinct samples or whether the same sample was measured repeatedly
☐	☒	The statistical test(s) used AND whether they are one- or two-sided<br>*Only common tests should be described solely by name; describe more complex techniques in the Methods section.*
☒	☐	A description of all covariates tested
☐	☒	A description of any assumptions or corrections, such as tests of normality and adjustment for multiple comparisons
☐	☒	A full description of the statistical parameters including central tendency (e.g. means) or other basic estimates (e.g. regression coefficient) AND variation (e.g. standard deviation) or associated estimates of uncertainty (e.g. confidence intervals)
☐	☒	For null hypothesis testing, the test statistic (e.g. *F*, *t*, *r*) with confidence intervals, effect sizes, degrees of freedom and *P* value noted<br>*Give P values as exact values whenever suitable.*
☒	☐	For Bayesian analysis, information on the choice of priors and Markov chain Monte Carlo settings
☒	☐	For hierarchical and complex designs, identification of the appropriate level for tests and full reporting of outcomes
☒	☐	Estimates of effect sizes (e.g. Cohen's *d*, Pearson's *r*), indicating how they were calculated

Our web collection on statistics for biologists contains articles on many of the points above.

Software and code

Policy information about availability of computer code

Data collection	Attester Software
Data analysis	GraphPad Prism (version 9.4.1), GATK (version 4.5.0.0), snpEff (version 5.2), Attester Evaluation software, TraceDrawer software (version1.9.1), BD FACSDiva (version 9.0.1) , FlowJo (version 10.8.1)

For manuscripts utilizing custom algorithms or software that are central to the research but not yet described in published literature, software must be made available to editors and reviewers. We strongly encourage code deposition in a community repository (e.g. GitHub). See the Nature Portfolio guidelines for submitting code & software for further information.

Data

Policy information about availability of data

All manuscripts must include a data availability statement. This statement should provide the following information, where applicable:

- Accession codes, unique identifiers, or web links for publicly available datasets
- A description of any restrictions on data availability
- For clinical datasets or third party data, please ensure that the statement adheres to our policy

Sequencing raw data in form of .vcf files is accessible on https://github.com/GMichlits/CCHFV-LDLR. The data generated in this study are provided in the Supplementary Information/Source Data file. Source data are provided with this paper.

Human research participants

Policy information about studies involving human research participants and Sex and Gender in Research.

Reporting on sex and gender	N/A
Population characteristics	N/A
Recruitment	N/A
Ethics oversight	N/A

Note that full information on the approval of the study protocol must also be provided in the manuscript.

Field-specific reporting

Please select the one below that is the best fit for your research. If you are not sure, read the appropriate sections before making your selection.

☒ Life sciences ☐ Behavioural & social sciences ☐ Ecological, evolutionary & environmental sciences

For a reference copy of the document with all sections, see nature.com/documents/nr-reporting-summary-flat.pdf

Life sciences study design

All studies must disclose on these points even when the disclosure is negative.

Sample size	For all experiments, 3 biological independent experiment for statistical relevance (One-Way ANOVA, student t-test, Kruskal-Wallis test with uncorrected Dunn's test)<br>For mice experiments, 12 mice per group, more than the number of mice usually used for these type of experiment (8 mice)
Data exclusions	No data excluded
Replication	3 independent experiments, consistent data between the experiments, all replication attempt were successful
Randomization	For in vitro study, randomisation not possible as either the same cells are treated differently, or the cells are genetically different: Thus, a random distribution of the samples was not possible.<br>For in vivo experiments, randomisation was not possible as mice in the 2 groups were genetically differents
Blinding	For all experiments (in vitro and in vivo), the samples curation was not blinded but the analysis were blinded (the experimentors that runned the experiments and the ones that analyzed the data were different, the samples not showing any information that could allow their identification during analysis).

Reporting for specific materials, systems and methods

We require information from authors about some types of materials, experimental systems and methods used in many studies. Here, indicate whether each material, system or method listed is relevant to your study. If you are not sure if a list item applies to your research, read the appropriate section before selecting a response.

Materials & experimental systems

n/a	Involved in the study	
☐	☒	Antibodies
☐	☒	Eukaryotic cell lines
☒	☐	Palaeontology and archaeology
☐	☒	Animals and other organisms
☒	☐	Clinical data
☐	☒	Dual use research of concern

Methods

n/a	Involved in the study	
☒	☐	ChIP-seq
☐	☒	Flow cytometry
☒	☐	MRI-based neuroimaging

Antibodies

Antibodies used	Anti-IFN type I receptor antibody (MAR1-5A3) (MAR1-5A3 [5A3]; Leinco Technologies, Inc.). VSV-M [23H12] antibody, EB0011,

Antibodies used	Kerafast. Goat anti-Mouse IgG (H+L) Cross-Adsorbed Secondary Antibody, Alexa Fluor™ 488, A11001, ThermoFisher. α-LDLR-PE Antibody, R&D Systems FAB2148P. ApoE antibody (Sigma, #AB947)
Validation	Validation of VSV-M antibody was done for western-blot by Kerafast. α-LDLR-PE antibody was validated for Flow cytometry against human LDLR by R&D system. The ApoE antibody was validated for neutralization in a previous study (Tréguier Y. et al, Virol J., 2022). The anti-interferon antibody was used in a previous study (Garrison A. et al, Plos Neg. Trop. Dis., 2017)

Eukaryotic cell lines

Policy information about cell lines and Sex and Gender in Research

Cell line source(s)	HEK293 (ATCC®, CRL-1573), HEK293T/17 (HEK293T, ATCC® CRL-11268™), A549 (ATCC® CCL-185) and Vero cells (ATCC® CCL-81). Haploid mouse Stem-Cells (mSCs, clone AN3-12) from IMBA, HepG2 (Abcam, AB275467), HepG2 ApoE KO (Abcam, AB280875), SW13 (ATCC, CCL-105), Bat Tb-1 Lu cells (ATCC, CCL-88)
Authentication	AN3-12 were authenticated by Haplobank (IMBA, Vienna). A549, HEK293, HEK293T and Vero cells were authenticated by STR profiling. HepG2 wt and ApoE KO were authentified by Abcam. Bat Tb-1 Lu were authenticated by ATCC.
Mycoplasma contamination	Cell lines were tested for mycoplasma contamination with negative results
Commonly misidentified lines (See ICLAC register)	No commonly misidifided cell lines was used in thi sstudy

Animals and other research organisms

Policy information about studies involving animals; ARRIVE guidelines recommended for reporting animal research, and Sex and Gender in Research

Laboratory animals	10 weeks old C57BL/6J mice (Charles River, Germany) and B6.129S7Ldlrtm1Her/J (LdLr KO), stock#002207, Jackson Laboratory, USA.
Wild animals	No wild animals were used in this study
Reporting on sex	Female mice. Sex was considered but female were choosen to avoid mice aggressiveness, more difficult to address in BSL4 conditions.
Field-collected samples	The study didn't involve fiels-collected samples
Ethics oversight	Stockholm Ethical Committee for animal research approved the research.

Note that full information on the approval of the study protocol must also be provided in the manuscript.

Dual use research of concern

Policy information about dual use research of concern

Hazards

Could the accidental, deliberate or reckless misuse of agents or technologies generated in the work, or the application of information presented in the manuscript, pose a threat to:

No	Yes	
☐	☒	Public health
☒	☐	National security
☒	☐	Crops and/or livestock
☒	☐	Ecosystems
☒	☐	Any other significant area

Hazards	Crimean-Congo Hemorrhagic Fever Virus

For examples of agents subject to oversight, see the United States Government Policy for Institutional Oversight of Life Sciences Dual Use Research of Concern.

Experiments of concern

Does the work involve any of these experiments of concern:

No	Yes	
☒	☐	Demonstrate how to render a vaccine ineffective
☒	☐	Confer resistance to therapeutically useful antibiotics or antiviral agents
☒	☐	Enhance the virulence of a pathogen or render a nonpathogen virulent
☒	☐	Increase transmissibility of a pathogen
☒	☐	Alter the host range of a pathogen
☒	☐	Enable evasion of diagnostic/detection modalities
☒	☐	Enable the weaponization of a biological agent or toxin
☒	☐	Any other potentially harmful combination of experiments and agents

Precautions and benefits

Biosecurity precautions	All our experiments involving VSV-CCH_G were done in biosafety level 2 laboratory and experiments involving CCHFV were done in biosafety level 4 laboratory in compliance with the Swedish Public Health Agency guidelines (Folkhälsomyndigheten, Stockholm).
Biosecurity oversight	Infections of cells are regulated the Swedish Public Health Agency SOPs reviewed by internal Biorisk committee. The internal Biorisk committe also reviewed and approved specifically the mice experiment described in this study
Benefits	Developement of antivirals to treat CCHFV infected patients.
Communication benefits	There is no risk communicating the information given in this manuscript

Flow Cytometry

Plots

Confirm that:

☒ The axis labels state the marker and fluorochrome used (e.g. CD4-FITC).

☒ The axis scales are clearly visible. Include numbers along axes only for bottom left plot of group (a 'group' is an analysis of identical markers).

☒ All plots are contour plots with outliers or pseudocolor plots.

☒ A numerical value for number of cells or percentage (with statistics) is provided.

Methodology

Sample preparation	Cells were dissociated with 500µl with TrypLE Express enzyme solution (Gibco) for 5 minutes and collected in FACS Buffer (D-PBS containing 5% FBS). After one wash with FACS Buffer, 10µl of α-LDLR-PE Antibody (R&D Systems FAB2148P) per 1E+6 cells was added and stained for 1h on ice in the dark. After one hour of staining, cells were collected by centrifugation and washed twice in FACS Buffer. Finally, cells were resuspended in 1ml of FACS Buffer
Instrument	Sorting was done on a FACS Aria III sorted. Flow cytometry was performed on a FACS LSR Fortessa instrument.
Software	Data was analyzed during sorting with BD FACSDiva (version 9.0.1) and re-analyzed for plotting data presented in this manuscript using FlowJo.
Cell population abundance	LDLR-negative, edited A549 cells that were sorted comprised 40.1% of single-cells (34.6% of total after cell, single-cell and LDLR-staining gating). LDLR-negative, edited Vero cells comprised 8.59% of single-cells (7.59% of total after cell, single-cell and LDLR-staining gating). LDLR-negative, edited NC8 iPSC that were sorted comprised 81.9% of single-cells (62.6% of total after cell, single-cell and LDLR-staining gating). The non-edited LDLR-negative single cell fractions of A549, Vero and NC8 iPSC were 0.92% 2.32%, and 47.9% respectively.
Gating strategy	Forward and side-scatter were used to define cells excluding debri and larger aggregates. Forward scatter area versus height was then used to define single cells. α-LDLR-PE-staining density was then plotted and LDLR-negative cells selected based on unmodified, stained cells. These cells were then sorted into a 96-well plate.

☒ Tick this box to confirm that a figure exemplifying the gating strategy is provided in the Supplementary Information.
